# Lectotypification of *Abies fanjingshanensis* (Pinaceae)

**DOI:** 10.3897/phytokeys.152.51494

**Published:** 2020-07-07

**Authors:** Yong Yang, Keith Rushforth

**Affiliations:** 1 State Key Laboratory of Systematic and Evolutionary Botany, Institute of Botany, Chinese Academy of Sciences, 20 Nanxincun, Xiangshan, Beijing 100093, China Institute of Botany, Chinese Academy of Sciences Beijing China; 2 The Shippen, Ashill, Cullompton, Devon, EN15 3NL, UK Unafiliated Cullompton United Kingdom

**Keywords:** *Abies fanjingshanensis*, China, gymnosperms, lectotypification, *Shenzhen Code*

## Abstract

The type collection of *Abies fanjingshanensis* W.L.Huang et al. contains four specimens in the Herbarium (GNUG). *Abies fanjingshanensis* is lectotypified here with *L.Yang 83-427* (GNUG0002022) under Art. 9.12 of the *Shenzhen Code*.

## Introduction

**Figure 1.**
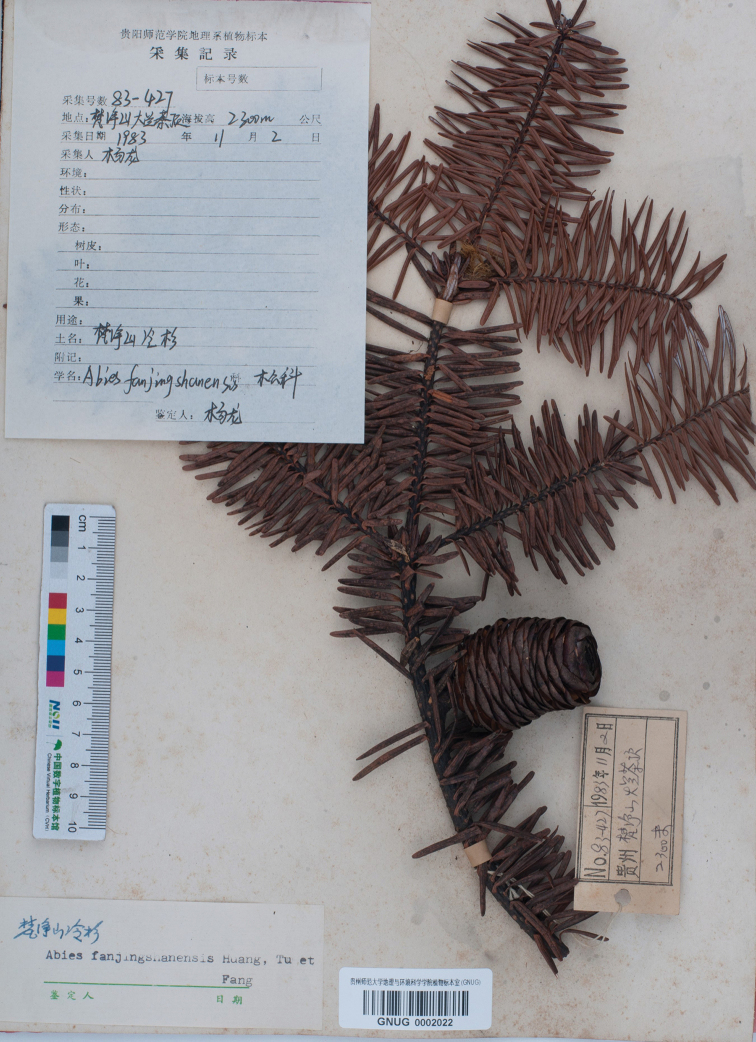
Lectotype of *Abies fanjingshanensis* (GNUG0002022), labelled with “*L.Yang* (杨龙)” as the collector.

**Figure 2.**
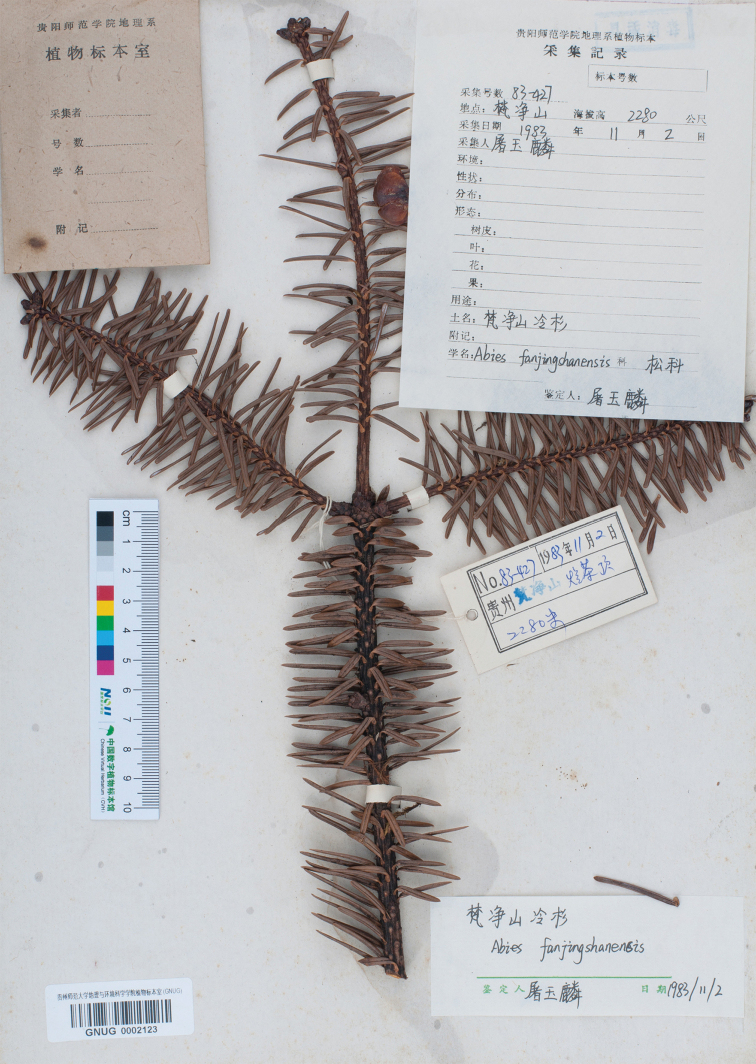
Isolectotype of *Abies fanjingshanensis* (GNUG0002123), labelled with “*Y.L.Tu* (屠玉麟)” as the collector.

**Figure 3.**
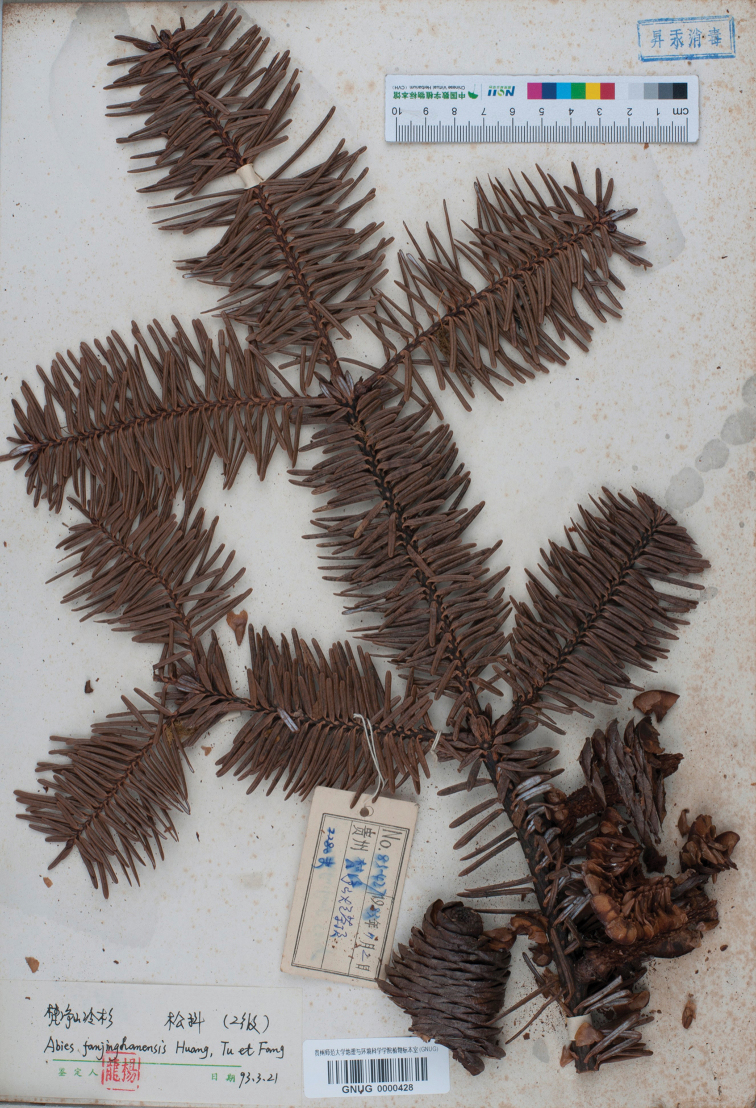
Isolectotype of (GNUG0000428), no collector is indicated on the collection label.

*Abies fanjingshanensis* W.L.Huang et al. of the Pinaceae is endemic to Fanjing Shan of Guizhou of south-western China (Huang et al. 1984; Fu et al. 1999). The type of the specific name is not clearly indicated. Huang et al. (1984) designated *L.Yang 83-427* (GNUG) as the type in the protologue. We found four specimens with the same collection number in GNUG, two of them [GNUG0002022 (Fig. 1) and GNUG0002122] are marked with “*L.Yang* (杨龙)” as the collector, the third one (GNUG0002123, Fig. 2) is marked with “*Y.L.Tu* (屠玉麟)” as the collector and the fourth one (GNUG0000428, Fig. 3) has no collector. All four specimens were identified as *Abies fanjingshanensis* and they match the characters of the species. It is reasonable to consider that *L.Yang* and *Y.L.Tu* are two collectors of a team because all four specimens were collected from the same locality *Fanjing Shan* on the same day 2 Nov 1983 and the two collectors are also the co-authors of the paper describing the new species. They may have collected the specimens together and gave the same number of the collection *83-427*, though they wrote different collector names on the collection notes. None of the four specimens is marked with “type” or equivalent words in Chinese. As a result, the type of *Abies fanjingshanensis* is not clearly indicated according to the existing original materials and we consider that the four specimens are the syntypes (Art. 9.5, Turland et al. 2018). Amongst the four specimens, two specimens are preserved with vegetative shoots having one or a few detached seed scales; one specimen (GNUG0000428) is a reproductive shoot having partially disintegrated female cones; and one specimen (GNUG0002022) is well preserved with a reproductive shoot having a good female cone. Thus GNUG0002022 is the most representative specimen and is one of the two annotated *L.Yang 83-427*. Accordingly, we designate it as the lectotype of *Abies fanjingshanensis* here under Art. 9.12 (Turland et al. 2018).

## Typification

### Abies fanjingshanensis

Taxon classificationPlantaePinalesPinaceae

W.L.Huang et al., Acta Phytotax. Sin. 22(2): 154 (1984)

02869D9B-0008-53F9-AF87-F4D0918E8962

≡ Abies fargesii var. fanjingshanensis (W.L.Huang et al.) Silba, Phytologia 68(1): 15 (1990).

#### Type. China.

**Guizhou** (贵州): Jiangkou (江口), Fanjing Shan (梵净山), northern slope along mountain ridge, alt. 2300 m, 2 Nov 1983, *L.Yang* (杨龙) *83-427* (lectotype, designated here: GNUG0002022; isolectotypes: GNUG0000428, GNUG0002123, GNUG0002122, PE00000459).

#### Note.

We found one specimen photo of the type collection *L.Yang 83-427* (PE00000459) in the Herbarium PE identified as *Abies fanjingshanensis* and labelled with “Isotypus” by L.K.Fu on 31 Jan 1989. We consider this specimen as the isolectotype.

## Supplementary Material

XML Treatment for Abies fanjingshanensis
